# A unique role for IL-13 in inducing esophageal eosinophilia through MID-1 and STAT6

**DOI:** 10.3389/falgy.2023.1248432

**Published:** 2023-11-06

**Authors:** Jason L. N. Girkin, Leon A. Sokulsky, Malcolm R. Starkey, Philip M. Hansbro, Paul S. Foster, Adam M. Collison, Joerg Mattes

**Affiliations:** ^1^ The Priority Research Centre for Healthy Lungs and The Asthma and Breathing Program, Hunter Medical Research Institute, University of Newcastle, Newcastle, NSW, Australia; ^2^ The Priority Research Centre, GrowUpWell^TM^ and The Asthma and Breathing Program, Hunter Medical Research Institute, University of Newcastle, Newcastle, NSW, Australia; ^3^ Department of Immunology and Pathology, Central Clinical School, Monash University, Melbourne, VIC, Australia; ^4^ Centre for Inflammation, Faculty of Science, School of Life Sciences, Centenary Institute and University of Technology Sydney, Sydney, NSW, Australia; ^5^ The Department of Respiratory and Sleep Medicine, John Hunter Children's Hospital, Newcastle, NSW, Australia

**Keywords:** eosinophils, eosinophilic esophagitis, allergy, interleukin 13 (IL-13), midline 1 (Mid1)

## Abstract

**Introduction:**

Eosinophilic esophagitis (EoE) is associated with allergen-driven inflammation of the esophagus and an upregulated Th2 cytokine signature. Recombinant interleukin (IL)-13 (rIL-13) administration to mice induces some of the hallmark features of EoE, including increased eotaxin expression and eosinophil recruitment. Inflammation in EoE has previously been shown to depend on the expression of TRAIL and MID-1, which reduced protein phosphatase 2A (PP2A) activity. The relationship between IL-13 and TRAIL signalling in esophageal eosinophilia is currently unknown.

**Objective:**

To investigate the interaction between IL-13-driven eosinophil infiltration and TRAIL or MID-1 in the esophagus.

**Method:**

We administered rIL-13 to wild type (WT), TRAIL-deficient (*Tnsf10*^−/−^) or STAT6-deficient (STAT6^−/−^) mice and targeted MID-1 with small interfering RNA.

**Results:**

rIL-13 administration to mice increased TRAIL and MID-1 expression in the esophagus while reducing PP2A activity. TRAIL deficient, but not STAT6 deficient mice demonstrated increased MID-1 expression and PP2A reduction upon IL-13 challenge which correlated with eosinophil infiltration into the esophagus. Silencing MID-1 expression with siRNA completely ablated IL-13 induced eosinophil infiltration of the esophagus, restored PP2A activity, and reduced eotaxin-1 expression.

**Conclusion:**

IL-13-driven eosinophil infiltration of the esophagus induced eosinophilia and eotaxin-1 expression in a STAT6-dependent and MID-1-dependent manner. This study highlights a novel mechanism employed by IL-13 to perpetuate eosinophil infiltration.

## Introduction

Eosinophil infiltration of the esophagus is associated with several inflammatory pathologies, including gastroesophageal reflux disease, parasitic infections, and eosinophilic esophagitis (EoE). EoE is an allergic phenomenon associated with food antigens, with a tendency to present primarily in young, atopic males (1–3). As a potential cause of dysphagia and food impaction, the treatments currently available to counter the associated morbidity are dietary restrictions, swallowed corticosteroid therapy and biologics such as interleukin (IL)-4 receptor antibodies [Dupiliumab] (4–7). With a rising prevalence in westernized societies (8), there is a greater need to elucidate the specific molecular pathways underpinning the pathogenesis to improve the quality of life of patients who do not respond to mainstream therapies.

The inflammation in EoE is associated with T helper 2 (Th2) driven eosinophilia via the cytokines IL-5 and IL-13 (9, 10). Both cytokines are elevated in patients with EoE in addition to *in vivo* studies modeling EoE. IL-5 plays a core role in eosinophilic trafficking to the esophagus, while IL-13 regulates the transcription of chemokines and cytokines involved in epithelial remodeling (e.g., Periostin, TGF-*β*) and inflammatory (CCL11, CCL24) cytokines through the transcription factor signal transducer and activator of transcription (STAT)-6 (11–15). Indeed, administering recombinant (r) IL-13 intratracheally to mice is itself sufficient to induce the hallmark features of EoE (13). IL-13-driven EoE was shown to be dependent on STAT6, IL-5, and eotaxin-1 (CCL11) using knockout mice, demonstrating that it plays an essential upstream role in inflammatory signaling in the esophagus (13). Despite this, other inflammatory pathways related to IL-13 signaling in EoE have yet to be investigated.

Previous studies on tumor necrosis factor-related apoptosis-inducing ligand (TRAIL) signaling in murine models of EoE have shown that inflammation and remodeling of esophageal tissue are dependent on the expression of TRAIL and downstream signaling through the E3 ubiquitin ligase Midline (MID)-1 (16, 17). TRAIL and MID-1 are known to augment allergen-induced inflammation (17, 18), but their relevance in specifically promoting IL-13-induced effector functions in EoE is unknown. In this study, we administered rIL-13 to TRAIL knockout (*Tnfsf10*^−/−^) mice and compared their inflammatory responses to mice that were STAT6 deficient (STAT6^−/−^). We also silenced MID-1 with small interfering RNAs (siRNAs) to wild-type (WT) mice to dissect further its role in IL-13-induced eosinophilic infiltration of the esophagus. We demonstrate that IL-13-mediated eosinophil infiltration depends on TRAIL and MID-1 and that MID-1-knockdown completely ablated eosinophilia and eotaxin-1 production, unlike TRAIL deficiency.

## Methods

### Mouse models of inflammation

Specific pathogen-free, 6–8 week-old, male Wide Type (WT), *Tnfsf10*^−/−^, and *Stat6*^−/−^ mice (on BALB/c background) were obtained from Australian Bioresources (Moss Vale, Australia). All animal experiments were approved by the Animal Care and Ethics Committee of the University of Newcastle. To induce inflammation of the esophagus, WT, *Tnfsf10*^−/−^, and *Stat6*^−/−^ mice were intranasally challenged with 15 μg of rIL-13 (in 35 μl of saline) under isoflurane anesthethesia administered in a supine position. Validated MID-1 siRNA (seq-5-AGAGUAAUCUCACCAAUCU-3) or control siRNA (seq-5-UGGUUUACAUGUCGACU AA-3) (3.75 nmol in nuclease-free water) (Dharmacon™) were administered 24 h before rIL-13 (Biolegend®) administration (19). Mice were sacrificed 24 h after the rIL-13 challenge via pentobarbitone overdose (Virbac), and the esophagus was collected for protein, histological, and mRNA analysis.

### Histological analysis of esophageal eosinophilia

Esophageal tissues were collected as described previously (16) and were stained for eosinophils (Congo-red). Eosinophil infiltration was determined by counting the number of eosinophils within 1 mm^2^ of the transverse esophageal section. Images were taken using the Aperio Slide Scanner (Leica Biosystems).

### RNA extraction and gene expression of esophageal tissue

Esophageal samples isolated from mice were homogenised in TRIzol® (Ambion, Life Technologies, Mulgrave, Australia) using the Tissue Tearor® rotor-stator. RNA was then extracted using chloroform phase-separation, followed by isopropanol precipitation ethanol wash and water-elution. RNA was reverse transcribed to cDNA using BioScript (Bioline, Alexandria, Australia) per the manufacturer's instructions. All genes (listed below) were quantified using qPCR SYBR® green (Life Technologies, Mulgrave, Australia) on the Eppendorf Realplex (Hamburg, Germany) platform. The primers were used at optimal melting temperature (60°C) and are listed in Table 1.

**Table 1:** Quantified genes and primer sequences.

β-actin	F 5-GACGGCCAGGTCATCACTATTG-3 R 5-AGGAAGGCTGGAAAAGAGCC-3
TRAIL	F 5-CCCTGCTTGCAGGTTAAGAG-3 R 5-GGCCTAAGGTCTTTCCATCC-3
MID-1	F 5-CACTCGCTGAAGGAAAATGACCA-3 R 5-AATCCAAGGCAAAAGTGTCAAA CG-3
CCL11	F 5-TTCTATTCCTGCTGCTCACGG-3 R 5-AGGGTGCATCTGTTGT TGGTG-3
POSTN	F 5-CGGAGAGCCAGTCATTAAAA-3 R 5-TGCAAGGTCTCTCCTGTTTC-3
STAT6	F 5-CTGGGAGTTCCTGGTCGGT-3 R 5-CTGTGGCAGAAAGTAGGGCAC-3

### Protein quantification and activity assays

Snap-frozen esophageal tissue was homogenised in Lysis Buffer consisting of IC#10 diluent (EGTA, EDTA, NP-40 alternative, HEPES, and water) and supplemented with Leupeptin (2.5 mg/ml), Pepstatin (1.25 mg/ml), Aprotinin (1.1 mg/ml), and PMSF. Assays were performed on clarified lysates. PP2A activity was determined by Active PP2A DuoSet IC activity assay as per manufacturer protocols (Active Motif). PP2A activity was normalised to Saline (SAL) vehicle controls. TRAIL protein expression was quantified through ELISA as per manufacturers protocols (R&D Systems).

### Statistical analysis

Statistical significance was determined between experimental groups using Student's *t*-tests (single comparison) or one-way ANOVA (Holm-Sidak) (multiple comparisons) in Graphpad Prism 6 (La Jolla, CA) as outlined in each figure legend. Data are presented as mean ± SEM.

## Results

### MID-1 is induced by IL-13 in WT and *Tnfsf10*^−/−^ mice, but not in *Stat6*^−/−^ mice

**Figure 1:**
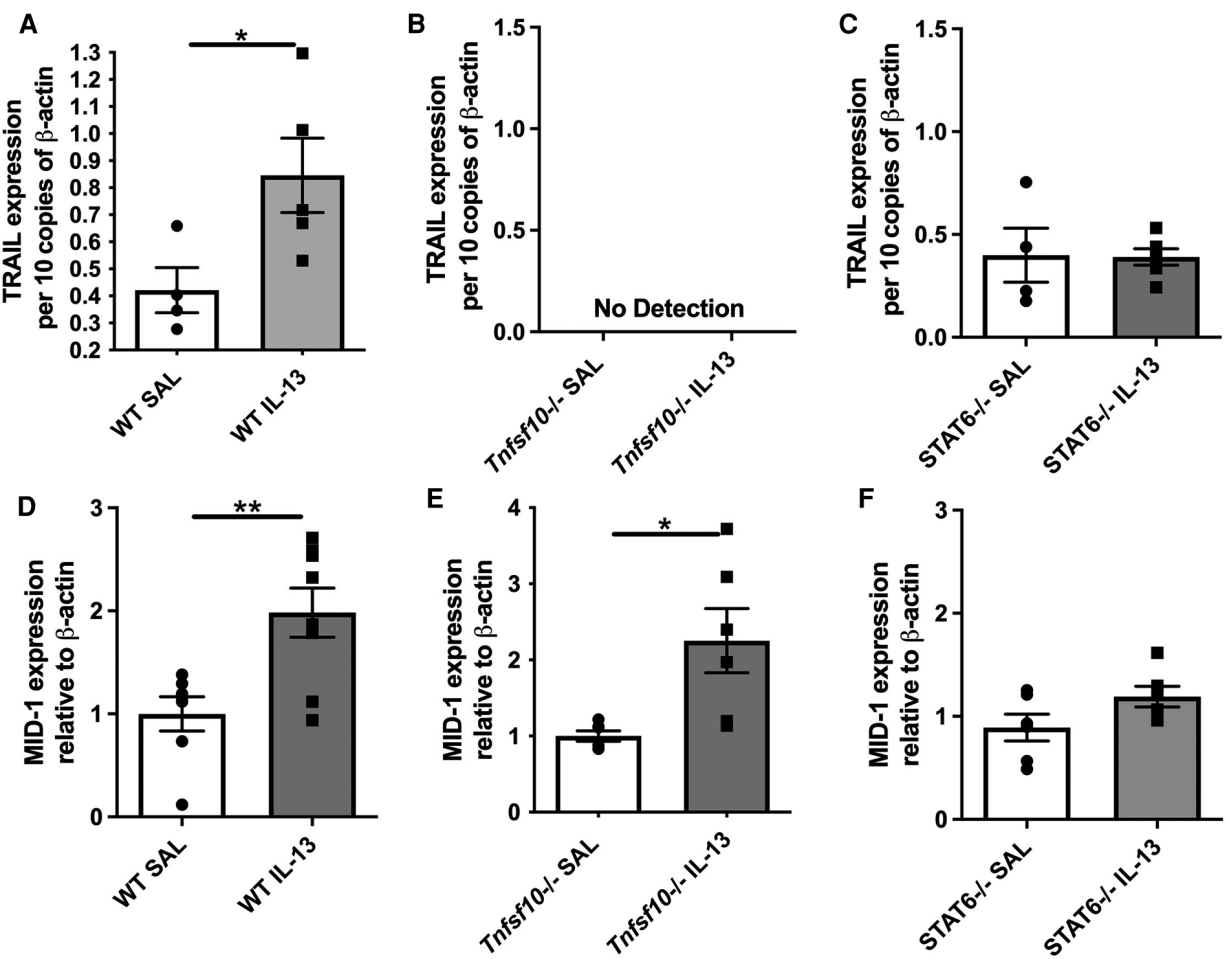
TRAIL, MID-1 expression, in the esophagus of WT, *Tnfsf10*^−/−^, and *Stat6*^−/−^ mice upon rIL-13 administration. TRAIL mRNA expression in (**A**) WT, (**B**) *Tnfsf10*^−/−^ and (**C**) *Stat6*^−/−^ mouse esophagi determined by qPCR, normalised to β-actin. MID-1 mRNA expression in (**D**) WT, (**E**) *Tnfsf10*^−/−^ and (**F**) *Stat6*^−/−^ mouse esophagi determined by qPCR, normalised to β-actin. Data expressed as mean ± SEM (*n* = 4–6). * = *P *< 0.05, ** = *P *< 0.01 as determined by Student's *t*-tests.

Our previous studies comprehensively outlined the role of TRAIL and MID-1 signalling axis in the propagation of allergic diseases of the airways and EoE models. To assess the role of the TRAIL and MID-1 signalling processes in the allergic mechanisms driven specifically by IL-13 in EoE, we assessed MID-1 expression downstream of IL-13 administration in *Tnfsf10*^−/−^ (TRAIL-deficient) mice and *Stat6*^−/−^ (transcription factor governing propagation of Th2-mediated allergic inflammation). Intranasal administration of rIL-13 to WT mice resulted in an upregulation of TRAIL protein (Figure 1A) and MID-1 mRNA expression (Figure 1D) in the esophagus. Interestingly, there was a significant increase in MID-1 expression in the *Tnfsf10*^−/−^ group in response to IL-13 (Figure 1D). As expected, *Tnfsf10*^−/−^ mice had no detectable TRAIL expression (Figure 1B). *Stat6*^−/−^ mice demonstrated baseline expression of TRAIL, however rIL-13 did not increase TRAIL above baseline in *Stat6*^−/−^ mice (Figure 1C). *Tnfsf10*^−/−^ but not *Stat6*^−/−^ mice had increased MID-1 expression upon IL-13 exposure (Figures 1E,F). Thus, IL-13-induced TRAIL and MID-1 expression is dependent on STAT6.

### TRAIL deficiency attenuates, while STAT6 deficiency ablates IL-13-driven eosinophilia in the esophagus

**Figure 2:**
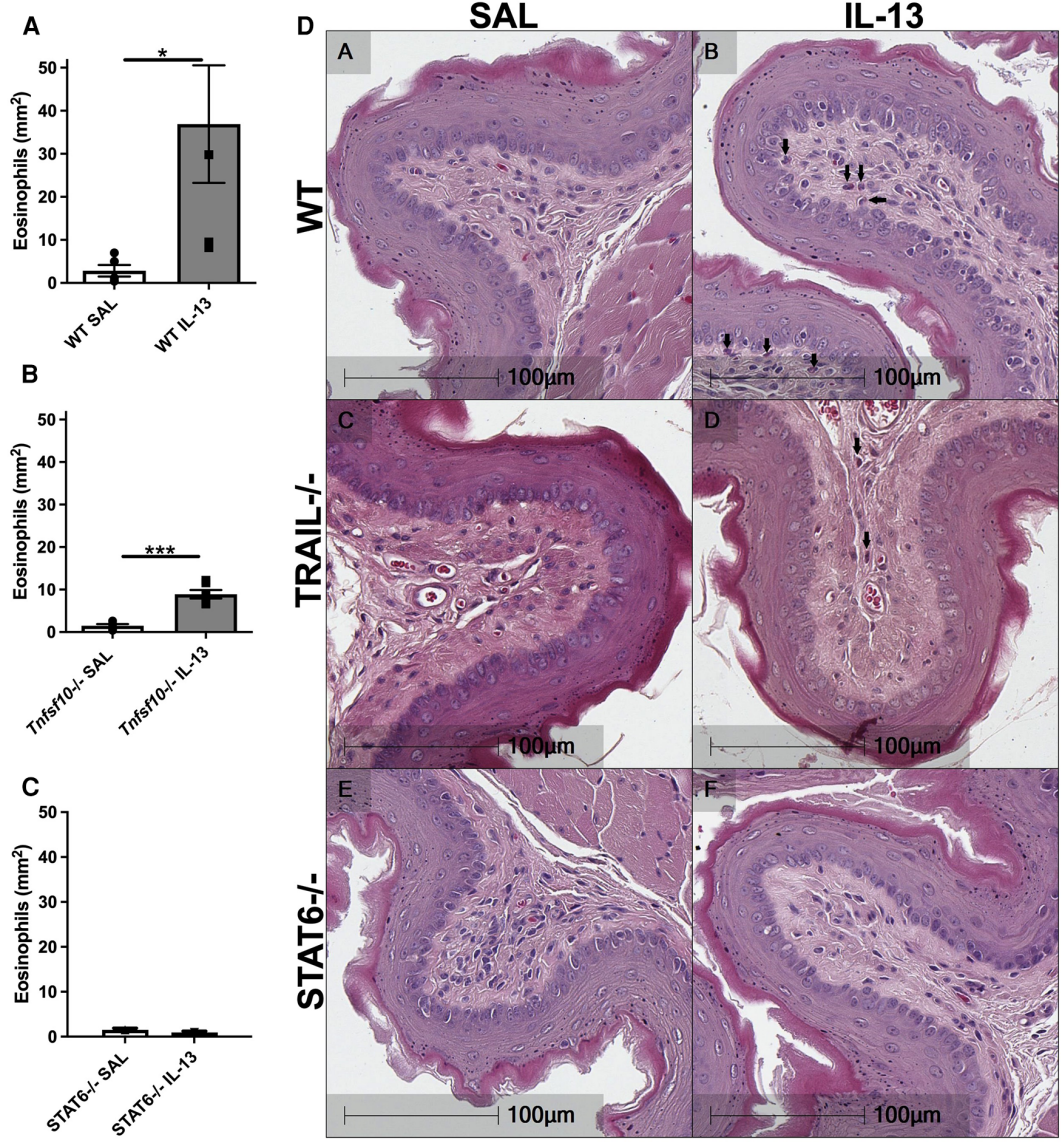
IL-13-induced esophageal eosinophilia is partially reduced in *Tnfsf10*^−/−^ mice and ablated in STAT6^−/−^ mice. (**A**–**C**) Average eosinophil counts in Congo-red stained traverse esophageal sections determined via light microscopy per mm^2^. (**D**) Transverse Congo-red stained esophageal pictographs (A = WT SAL, B = WT IL-13, C = *Tnfsf10*^−/−^ SAL, D = *Tnfsf10*^−/−^ IL-13, E = STAT6^−/−^ SAL, F = STAT6^−/−^ IL-13). Data expressed as mean ± SEM (*n* = 3–6). ** = *P *< 0.01, *** = *P *< 0.001 as determined by Student's *t*-tests.

Eosinophils are the hallmark effector cell in EoE and are recruited downstream of IL-13 signalling. Therefore congo-red stained transverse esophageal sections were analysed using light microscopy in our experiments. The number of eosinophils (per mm^2^) was determined in WT, *Tnfsf10*^−/−^and *Stat6*^−/−^ mice upon IL-13 exposure (Figures 2A–D). While rIL-13 administration resulted in a marked increase of eosinophil infiltration in WT mice (Figure 2A), eosinophil recruitment was attenuated in *Tnfsf10*^−/−^ mice (Figure 2B), and completely ablated in *Stat6*^−/−^ mice (Figure 2C).

### IL-13 upregulates eotaxin-1 and periostin in the esophagus of WT and *Tnfsf10*^−/−^ mice, but not in *Stat6*^−/−^

**Figure 3:**
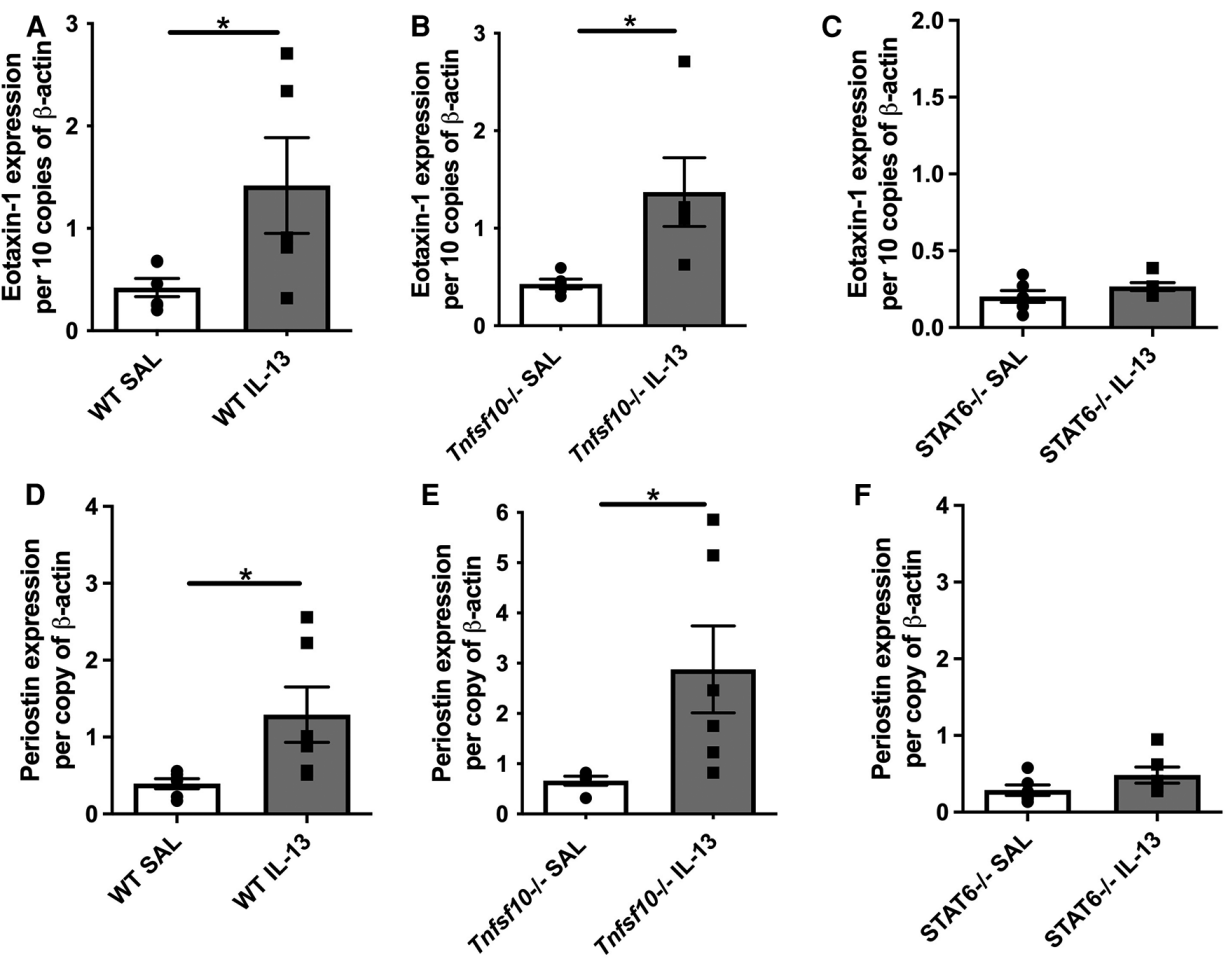
TRAIL deficiency does not reduce IL-13-induced eotaxin-1 and periostin mRNA expression in the esophagus, unlike STAT6^−/−^ mice. (**A–C**) Eotaxin-1, and (**D–F**) periostin mRNA expression in mouse esophagi determined by qPCR, normalised to β-actin. Data expressed as mean ± SEM (*n* = 5–6). * = *P *< 0.05 as determined by Student's *t*-tests.

Eotaxin-1 and periostin are effector cytokines and immune correlates of disease in EoE. We therefore sought to determine whether their expression induced by IL-13 is dependent on TRAIL/MID-1 and STAT6 signalling. Both WT and *Tnfsf10*^−/−^ mice significantly increased eotaxin-1 expression after rIL-13 administration (Figures 3A,B), which was not observed in *Stat6*^−/−^ mice (Figure 3C). Similarly, periostin expression was significantly upregulated in rIL-13 administered WT and *Tnfsf10*^−/−^ (Figures 3D,E) but not *Stat6*^−/−^ mice (Figure 3F). In summary, IL-13 induced expression of eotaxin and periostin is STAT6 but not TRAIL-dependent.

### MID-1 siRNA administration restored PP2A activity but not TRAIL or STAT6 expression after esophageal IL-13 exposure

**Figure 4:**
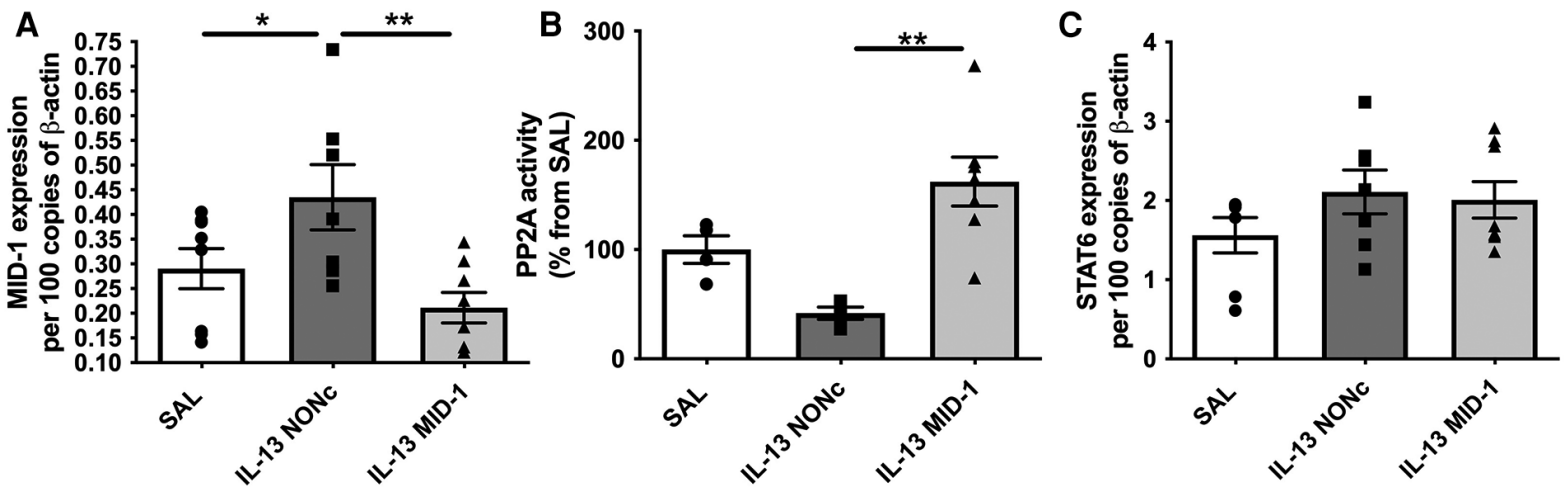
Silencing MID-1 before IL-13 administration restores PP2A activity in the esophagus but does not alter TRAIL and STAT6 expression. (**A**) MID-1 mRNA expression in mouse esophagi was determined by qPCR, normalised to β-actin, in saline exposed (SAL) mice, or nonsense control siRNA (NONc) and MID1-silencing siRNA (MID-1) IL-13 exposed mice. (**B**) PP2A activity measured in mouse esophagi normalised to esophageal tissue weight and respective SAL control (%). (**C**) STAT6 mRNA expression in mouse esophagi determined by qPCR, normalised to β-actin. Data expressed as mean ± SEM (*n* = 4–8). * = *P *< 0.05, ** = *P *< 0.01. as determined by one-way ANOVA (Holm-Sidak).

The MID-1 signalling pathway is thought to propagate inflammation by causing polyubiquitination and degradation of protein phosphatase 2a (PP2A). PP2A is a constitutive negative regulator of inflammation. Reduced PP2A resulted in inflammation through subsequent increase ERK-1/JNK inflammatory signalling. In order to suppress MID-1 expression locally we administered MID-1 targting siRNA in the esophagus. As expected MID-1 expression was significantly reduced compared to the nonsense (control) siRNA (NONc) control (Figure 4A). Further, the reduced MID-1 expression resulted in increased PP2A activity (Figure 4B). The lack of difference between saline and NONc control siRNA IL-13 exposed mice remains unexplained but could be related to unspecific siRNA effects. STAT6 expression was not altered by MID-1 inhibition (Figure 4C) indicating that STAT6 is upstream of MID-1 mediated suppression of PP2A during IL-13-driven inflammatory signalling in the esophagus.

### MID-1 silencing ablated Il-13-driven eosinophilia and eotaxin-1 expression in the esophagus

**Figure 5:**
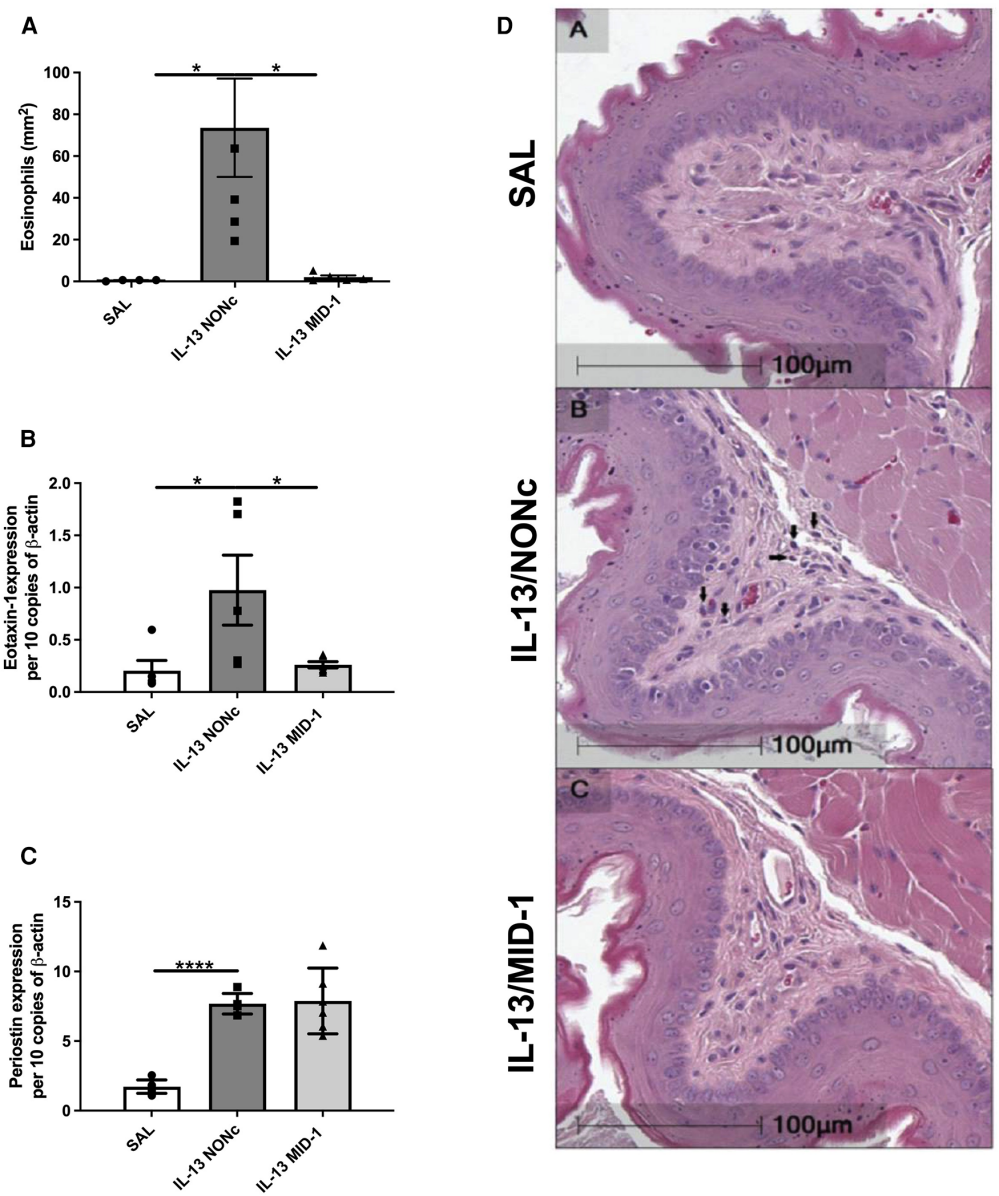
Ablation of IL-13 induced eosinophilia and eotaxin-1, but not periostin, in MID-1 siRNA-treated mice. (**A**) Average eosinophil counts in Congo-red stained traverse esophageal sections determined via light microscopy per mm^2^. (**B**) Eotaxin-1 and (**C**) periostin mRNA expression in mouse esophagi determined by qPCR, normalised to β-actin. (**D**) Transverse Congo-red stained esophageal pictographs (A = SAL, B = NONc siRNA/IL-13, C = MID-1 siRNA/IL-13). Data expressed as mean ± SEM (*n* = 4–6). * = *P *< 0.01, **** = *P *< 0.0001 as determined by one-way ANOVA (Holm-Sidak).

Targeting MID-1 using siRNA (as above) prevented IL-13-induced eosinophilic infiltration compared to the NONc control (Figures 5A,D). There was also a significant reduction of eotaxin-1 mRNA expression in the esophagus (Figure 5B). No changes in periostin expression were observed (Figure 5C) indicating that IL-13 driven eosinophil recruitment, could be dependant on eotaxin-1 (not periostin), and that eotaxin-1 is downstream of MID-1.

## Discussion

Recombinant IL-13 has been shown to induce some hallmark features of experimental EoE in animal models. For instance, IL-13 regulates the expression of eosinophil-attracting chemokines in esophageal epithelial cells (14). In allergen driven *in vivo* models, we have shown that IL-13 production was dependent on the expression of TRAIL and MID-1 (16, 17). However, it remained unclear whether IL-13 alone can propagate eosinophilic inflammation through the MID-1 and PP2A siginalling pathway. Here, we show that intranasal administration of rIL-13 to *Tnfsf10*^−/−^ mice resulted in reduced eosinophilic infiltration compared to wildtype mice without effecting eotaxin-1 expression. This may be explained by the reduced levels of other cytokines regulating eosinophilic chemotaxis in *Tnfsf10*^−/−^ mice described earlier (18). Interestingly, targeting MID-1 with siRNAs resulted in complete ablated IL-13-mediated EoE features, unlike seen in *Tnfsf10*^−/−^ mice. These findings indicate that IL-13 propagated EoE features through MID-1 and although TRAIL does play an important a role in EoE, it is partly dispensible when recombinant IL-13 is directly administered.

MID-1 siRNA ablated IL-13-induced eosinophilic infiltration in the esophagus to levels comparable with IL-13-challenged *Stat6*^−/−^ mice. MID-1 silencing also reduced eotaxin-1 expression but not periostin expression. Thus MID-1 and STAT6 are essential for IL-13-induced eotaxin-1 expression and eosinophil recruitment, even though a MID-1 independent pathway mediates periostin expression upon IL-13 signalling. These results define MID-1 as an integral component of the IL-13-mediated, STAT6-dependent proinflammatory effector pathway in the esophagus.We have previously shown that IL-13 is required for TRAIL-induced eosinophil recruitment of the lung (18). Our new data indicate that TRAIL can partially mediate some of the IL-13-driven EoE features, but is redundant following rIL-13 instillation.

Upregulation of MID1 following rIL-13 exposure was reduced in *Tnfsf10*^−/−^ mice but completely abolished in *Stat6*^−/−^ mice. This suggests that IL-13 induced MID1 expression can occur in a Stat6-dependant manner. Furthermore, MID-1 siRNA ablated both eotaxin-1 expression and eosinophilia after rIL-13 administration, which indicative that IL-13 induces eosinophilic infiltration through MID-1 signaling. We therefore infer that IL-13 propagates hallmark EoE inflammatory features (e.g., eosinophil recruitment and inflammatory mediators) through Stat6 and then MID-1.

In our previous works, IL-13 and STAT6 were dependent on TRAIL and MID-1 expression in allergen-driven EoE (16). Now, this current study demonstrates that rIL-13 can induce TRAIL and MID-1 expression in the esophagus, indicating that IL-13 is not merely to be an effector molecule downstream of TRAIL-induced inflammation. It is also a modulator of MID-1 mediated inflammation. Taken together, these studies could indicate a positive feedback loop between IL-13 and TRAIL in the perpetuation or progression of EoE. In addition, intratracheal rIL-13 administration was identified as a critical driver of Th2-driven inflammation of the esophagus dependent on eotaxin-1 and STAT6 (13) which complements and reinforces our findings. Our previous works showed that eotaxin-1 and STAT6 were regulated by TRAIL (16, 17). Here we identify, for the first time, that IL-13 can perpetuate eosinophil infiltration through MID-1.

We propose that IL-13, signaling through the E3 ubiquitin ligase MID-1, modulates PP2A activity in a STAT6-dependent manner. Targeting IL-13 with biologics is now a therapeutic option for EoE patients (20). Our data highlights MID-1 as a new, potentially critical element of IL-13 induced EoE pathology, and as a new therapeutic target. Future studies are required to completely characterize MID-1 regulated signaling pathways relevant to the development of inflammation employing for instance RNA sequencing and proteomics.

## Data Availability

The raw data supporting the conclusions of this article will be made available by the authors, without undue reservation.
